# Chapter II: Diarrhœa

**Published:** 1883

**Authors:** 


### CHAPTER II.

### DIARRHŒA.

The affections which pass under the above denomination are exceedingly various in their causes, characteristics, importance, and curative relationships. The object of the present paper is not to present an exhaustive view of the subject in these particulars, but to give such an analysis of them as will facilitate their successful treatment, especially by those who are comparatively inexperienced in the practical duties of our profession. To such, in the beginning, one disease is very like every other which is called by the same name. It is only after many painful buffetings and sore disappointments that he comes to suspect that *names* are not *things*, and finally to see clearly that many conditions, called by the same name, are so different in their characters as to be essentially different affections.

In his elementary education he has been taught to treat *names*, or, as it has been expressed—with a seeming scientific wisdom—"*diseases;*" and for such and such *diseases* he is to do so and so, and he does it with conscientious carefulness and confident expectations of the promised result—a cure. This does not always follow, and repeated disappointments, by and by, remove the delusion derived from his master. Then he has one of two courses before him: either to settle down into professional skepticism, or, if he be able and willing, to work out for himself a better faith, which shall rest on ascertained truth, can bear him up in his toils, and justify his confidence by a reasonable success. The first expresses the history of many earnest and honest young physicians who would have been lights in the world if they had not, in the outset, adopted a false faith and attempted to practice upon a false principle. Upon a discovery of this error, they jump to the other and greater—that there are no true principles in medicine. To them, all are equally false. They become the Micawbers of the profession—the expectants. He who will honestly follow the second course will soon discover that the great duty of his life has been much misrepresented and misconceived. He is not to treat *diseases*, *names*, *things*—imagined somethings, which have somehow found their way into live humanity and made it suffer and perhaps are bringing its existence into peril. He will realize after a while, though not perhaps immediately, that that with which he has to do is not an entity which somehow has effected bodily entrance into his poor patient, like demons into the possessed, though all surrounding and sympathizing friends regard the matter somewhat so, and fully expect him to exorcise the intruder.

He will find that what he has to treat is the patient himself, and not an extraneous intruder within him. Till he is fully master of this fundamental principle, and in its light has abandoned all thought of treating diseases as something distinct from the patient, he is in no way qualified for the practical duties of the high calling to which he has devoted himself. He is to treat sick men, women, and children—*patients*, and not *diseases*. It may be late in the history of practical medicine to inculcate this principle. No doubt it is. But its truth and importance will admit of no longer postponement. Delay has neither diminished its truth nor value. Rightly appreciated, it at once disposes of that complacent piece of arrogance which has asserted a superior science in the old school of our profession, because that school treats *diseases*, while ours treats only *symptoms*. This principle clearly asserts the fact that true science treats patients, and *not* diseases. It may be, and probably is, true that the old school treat diseases, or attempt this, and think they succeed, but it is not true of ours that when we rightly appreciate and practice its doctrines, "we treat only symptoms." If there be one more false than another among the slanders by which the homœopathic school has been assailed, it is this. When rightly practiced, Homœopathy is cognizant of all those facts in relation to the patient which constitute the difference between the man sick and the man in health.

What, then, is disease? It is only a *condition*, not a *thing*. It is the sum of whatever modified actions of the vital forces, by which that harmony is lost which conserves the integrity of all parts of the living organism. A knowledge of the totality of these modifications constitutes the science of *pathology*. A knowledge of the results of these modifications, of their products and changed tissues, constitutes the anatomy of pathology. To call these *the disease*, as many have done, is no less absurd than to call the residual contents of the intestines *digestion*. An analysis of these modifications, a resolution of them into their elements, is the first step in the process of all right prescribing. By this proceeding only can the elements of a given case be exposed, so that it can be seen what is individual in it is characteristic of this member of a class, and these elements be separated from those which are generic, *i. e.*, belonging equally to all members of that class; a distinction without which a life of prescribing for the sick is a life in the dark.

If this be so, it may be asked, what is the value of a nomenclature of diseases? Why attempt to name them at all? The answer is, It is a convenience in the expression and interchange of ideas—indispensable, if you please. By the name is simply meant to announce a group of phenomena which are found in a given class of affections and which belong equally to each member of the class, and which distinguish it from all other classes. Thus, by the term at the head of this article is meant to express in one word the following group:

Frequent discharges from the intestines of feculent, secreted, or undigested matter. It may be of either alone or of either two or all of them mixed. We propose in this paper a brief analysis of these affections, in order to their more ready homœopathic treatment.

In order to this we observe that these frequent discharges are farther diversified by the following peculiarities, which are important to be noted in the selection of a remedy for their cure. They are *painful* or *painless*. The secreted discharges are *mucous*, *serous*, or *purulent*. These and the feculent are further characterized by difference of *color*, as *black*, *brown*, *gray*, *green*, *red*, *white*, and *yellow*; and by difference of odor, as of *spoiled eggs*, *putrid*, *acid*, etc ; and also by difference of time and circumstance by which the affection is either excited or aggravated.

The first element of the above analysis, the painful diarrhœas, are related to curative drugs by this quality, in different degrees; *i. e.*, some drugs produce diarrhœas with intense pain, others with less severe, and others again with pains still more moderate. These distinctions are to be noted in selecting the curative drugs. Thus, for those with severest pains we have Ars., Coloc., Jalap., Rheum, Rhus tox., Senna. For the second class Bryonia, Carbo veg., Caps., Cham., Dulc., Merc., Nux vom., Petrol., Puls., Sulphur and Verat. For the less painful, Agar., Aur. mur., Anac., Asaf., Asar., Spig. The painless diarrhœas are related to drugs also in different degrees, *i. e.*, some medicines are more and some less characterized by them, and so are more or less frequently required for their cure. There cannot be, as in the previous class, degrees of this peculiarity, but only a difference in the degrees of tendency of the drugs to produce this kind of affection. In the first rank we may place Ars., Ferr., Hyos., Lyc., Phos., Phos. acid., Stann. Second, Bell., Cham., Chel., Chin., Opium, Plat., Sulph. Third, Borax, Bov., Calc. c., Carb. an., Cocc., Dulc., Graph., Hell., Ign., Laur., Mag. c., Merc., Nit., Nit. acid, Puls., Rhod., Rhus tox., Sec. corn., Verat., Zinc.

In a given case to be prescribed for, it is ascertained to be painful or painless, and after reference to the list of drugs and to their classes, as above, how are we to determine the one required for the cure? By the continuance of the analysis to the other elements of the case. And, first, consider the character of the pain, and, second, the locality of it. Pains with the diarrhœa may be burning, cutting, constricting, pressing, dull, excoriating, etc. It would exceed the limits proposed to go into a statement of all the medicines which produce these different pains, with their affections of the bowels. It may be sufficient for the present purpose to point to them generally. Thus, diarrhœas with *burning* pains, Ars. and its cognates. But Ars. will not cure all cases with such pains. Neither is it always the best remedy for some cases which perchance it may cure ultimately. If, for example, the *burning* be confined to the lower part of the rectum, and is accompanied by throbbing and sense of excoriation, with pain in the back, continuing after the evacuation, Capsicum is the remedy, and Arsenic will probably fail to relieve. This very familiar example is given to show the necessity of carrying the analysis of the leading features of cases forward to all their relations if we would secure the best possible results of our prescriptions with certainty. We can never neglect this with safety to our patient or with honesty of practice.

With *cutting* pains, Coloc. and its cognates. With Coloc. the pain is relieved by the evacuations, is very sharp, doubles the patient up, is accompanied with outcries, and often with slight nausea; the pains are more paroxysmal than with *Ars.*, which in relation to cuttings in the intestines it much resembles, and are rather of a neuralgic than inflammatory character. With *constricting* pains, Plumb. and its cognates. With this remedy, and also with Podophyllum there is not only a sense of constriction, but a real retraction of the parieties of the abdomen. With pressing or squeezing pains, Nux vom. and its cognates. With this remedy the pressure is more in the upper part of the abdomen and sides. With pain like *excoriation*, Sulphur and its cognates, as Ars., Bell., Nux vom., etc.

The *locality* of the pain is important in this investigation. Different drugs affect different portions of the alimentary track painfully. Some, as Senna and Jalap, attack the upper portion, or the small intestines chiefly; others, as Aloes, Nux vom., Caps., Merc., the larger; while still others, as Ars., Colch., Verat, etc., affect the whole track. A careful attention to the pathogenesis of the drugs will enable the student to ascertain the peculiar local action of each, and to avail himself of this knowledge in his attempts at specific cures of diarrhœas. This study he cannot omit, if he is ambitious of the best success in his practice. Whether the remedies named above or either of their cognates are to be selected in a given case, is to be decided after having reference to the above peculiarities of the pain, by consideration of the remaining elements of the analysis. And of these, the next to be considered is the character of the expelled contents of the intestines. They may be *feculent, mucous, serous*, or *purulent.*

For *feculent* diarrhœas we have Aloes, Pod., and Rheum.

Aloes has both yellow and brown color.

Pod. yellow and dark green. The diarrhœas of this remedy are often accompanied by *prolapsus ani*, especially in children, and for this complication it is one of our best remedies.

*Rheum*—Feces mixed with green slime.

*Mucous* diarrhœas may be *brown, green, red, white*, or *yellow.*

For the *brown* we have Ars. and Nux v., the Ars. being characterized by a mixture of mucus and feces; that of Nux v. is brown, offensive, and slimy.

*Green* mucus has Ars., Amm. mur., Canth., Cast., Laur., Mag. c., Merc., Nux v., Puls., Rheum, and Tabac.

The practitioner will use great caution in prescribing for this class of diarrhœas, in his search into the constitutional and related symptoms of his cases, if he would avoid disappointment and doing his work twice or thrice over. This is especially to be observed in the case of the two remedies in the class more frequently prescribed than any others, viz.: Ars. and Merc. The habit of hasty, and therefore, careless, prescribing, so easily contracted and so common, may be a sufficient apology for saying that cases requiring either of these drugs will certainly disclose other and characteristic symptoms of the one to be selected if the examination be diligent, careful, and intelligent. This is not only true of *Ars.* and *Merc.*, but of each of the other members of the class, and the observation may be extended to every other symptom of every other class of this disease. No case is made up of one symptom, however marked or important, and it is not unfrequent that the controlling characteristic of a case—that element more decisive than any one other of the selection of the curative drug—is just that which carelessness and haste are very likely to overlook. These observations are made in connection with the two named remedies, because failure with them, in this class of diarrhœas, is too common.

Diarrhœas of *red* mucus are related to Merc., Rhus, Sil., Sulph. The distinction of these four remedies in their application to *red mucous* diarrhœas is not difficult. Merc. has plain red mucus, with the characteristic pain and tenesmus of Mercurial diarrhœas; Rhus has a mixture of blood and slime, with red and yellow mucus, and all rather thin; Sil. has red mucus *with the stool* of which it may or may not constitute the major part; Sulph. has red mucus *with fever*, loss of appetite, and cutting pains in the bowels.

*White* mucous diarrhœa has Cham., Dulc., Phos., Pod., Puls.

After a proper consideration of the general symptoms, if there be doubt as to which of these medicines is required for a given case, it may help to remember that the affection requiring Cham. is painful, and is more frequent in the affections of childhood than of adult life. That for Dulc. is attended with prostration of strength; with Puls. the mucus is *acrid;* with Pod. the diarrhœa occurs for the most part mornings or forenoons, the pains in the abdomen and back are worse *during* the evacuation and continue *after*. The discharges are excited by eating and drinking. With Puls. the pain is *before* the evacuation, is likely to be attended with much rumbling of the bowels, and the peculiar disposition of mind so characteristic of this drug.

*Yellow* mucus has Dulc., Pod., Rhus, Sulph. acid.

Dulcamara is especially indicated where the color of the slimy stools frequently alternates between green, white, and yellow, and the desire to evacuate is attended with *nausea*, or where the attack is the result of *chill.* Podophyllum is called for when the yellow color is *dark* and the evacuation has the *odor of carrion;* with Rhus the stool is *mixed* sometimes with *blood* or *red slime*, or consists of bilious-looking matter, and all *very thin.* In Sulph. acid the stools are like chopped mucus, saffron yellow and stringy. The above examples of the first step in the analysis of the evacuations in diarrhœa are given not as instances of the completed process in this first step, but only as illustrative of the mode of procedure in relation to the two elements of *nature* and *color.* It is not enough that the discharge be *mucus*, nor that it be also *green* or *yellow*, to decide the choice of the curative. We must know more, even *all* the peculiarities of the evacuations, and much more than this, as will be seen as we advance. For it is not to be forgotten that the object is not so much to put into the hands of practitioners a "short and easy method" by which all cases can be cured with little or no labor on their part, as to point out the way in which they may obtain a success worthy of the system of medical science we profess to practice. If our object were otherwise, its folly would be rebuked by the first glance at the nature of the case. The object of the practitioner is to find in the pathogenesis of a drug the simillimum of the sum of aberrations of the vital forces in a given case from that state of harmony we call health. These are so various in their nature, importance, and combinations, and so numerous withal, that any attempt to make the labor of an exact practice "short and easy," *i. e.*, to find this simillimum, could hardly be otherwise than absurd in the extreme. The number of possible elements in any case is great, and may be very great, and the variety of combinations they are susceptible of is scarcely less than infinite. It is evident, hence, that there can be no such method of ascertaining from the scarcely less infinite record of facts of which our materia medica is composed, the parallel of a given case, except by the exercise of patience and diligence. How to direct these, by pointing the way through the medium of the more general and common elements of cases, is our present endeavor, and in its furtherance we consider:

*Watery* diarrhœas, which are found to be *black, green, gray, yellow;* and nearly allied to these, are the *brown fluid* and *black fluid.*

*Black* watery diarrhœas have Ars. and China. At this point these remedies are in close resemblance; so near that from the *black water* alone, no man can tell whether the one or the other is required. But a careful consideration of the other elements will render the selection easy. As a general truth, the prominent effects produced by Ars. are characterized by *violence,* and, among them, this is eminent in its effects on the alimentary canal. Now, the difference between these members of this class of diarrhœas which decides the choice of the remedy between Ars. and China is in the violence of the symptoms to be considered. The pain, burning, restlessness, prostration, cold sweating, etc., are all greater in cases requiring Ars.

*Black* fluid diarrhœas have Stann. and Ars.; the latter burns like fire.

*Brown* fluid have Arn., Asaf., Graph., Magn. c., Nux v., Psorin., Squill.

The discharge which in this class is peculiar to Arn. resembles yeast or lees of beer. In Asaf. the evacuation is *extremely* and *nauseatingly* offensive. In Graph. it is in part made up of half digested substances, and of insupportable fetor. Magn. c. has a *liver-brown* colored discharge, with tenesmus, followed by burning in the anus. It is characteristic of this, and all the varieties of diarrhœa produced by Nux v. that the evacuations are *small in quantity,* they are more frequent in the morning and after eating, and are for the most part accompanied by tenesmus and pain in the *back* of a *drawing* character. In this variety there is also smarting and burning in the anus. In Psorin. it is *dark* brown, very thin, and offensive. In Squill. it is *dark* brown or even *black,* slimy, very offensive, and *ejected in frothy bubbles,* with flatulence, and sometimes with ascarides and whitish shreds.

*Green* watery diarrhœas are met by Cham., Gratiola, Magn. c., and Sulph. acid. It may not be out of place to remark here, in relation to this class of the affections under consideration, that it is perhaps more frequently misunderstood and, therefore, more frequently wrongly treated than any other. Much of the disappointment necessarily consequent on such a course may be avoided by remembering, in the first place, the too often overlooked, but vastly important, necessity of making the *first* prescription a *right* one; and in the second, that Ars. does *not* cure this variety of diarrhœas. If there be any exception to this, they are cases where the remedy accomplishes the result by virtue of its characteristic relationship to the *constitutional* symptoms of the case. Of this we may have more to say hereafter. It has not been an unfrequent experience of the writer to see cases of this variety of diarrhœa, in consultation, and among them, the most intractable to treatment have been those which had Ars. as their *first* medicament. That this has often proved a serious embarrassment to the subsequent successful management of these cases he has no doubt. The frequency of this false prescription is, perhaps, explained by the force of habit. Ars. cures so many forms of diarrhœa that the frequent demand for its use creates a kind of *habit* of prescribing it. Against this we protest.

The cases for Cham. are for the most part those of early childhood, during the process of teething and from taking cold. The green watery passages are often mixed with feces and mucus. The *green* and *frothy* evacuations of Grat. may be watery or thin fluid or slimy. It is a remedy worthy of more attention, in diarrhœas, than it has generally received, especially with those of children, in the summer season. Those of Magn. c. are preceded by pinching pains in the bowels, especially in the right side, with distended abdomen, and mostly in the forenoon, and may be both sour smelling and frothy. Sulph. acid is frequently the right remedy in this variety of diarrhœa. In the absence of the characteristic signs of the other medicines, it may be given in preference, and especially if there be great prostration of strength with irascibility of temper.

*Yellow* watery diarrhœas are met by Ars., Chin., Grat., Hyos. They may be found in the pathogenesis of a few other drugs, but the four above named are the principal remedies, and rightly used will succeed with most of these cases. Here, as in the *brown* variety, Ars. and Chin. are near together. Both have attacks more frequent at night and after eating and drinking, with great prostration. But Ars. has tenesmus, Chin. has not. Ars. has thirst with diarrhœas, Chin. has not. Ars. has a painful constriction above the anus, extending to the loins. With Ars., in this variety, the discharges are *small*, while in many others they are copious. But if, as is not at all unlikely, the peculiar and distressing *restlessness* so characteristic of Ars. be present in any case, there need be no hesitation in the choice between the two drugs. The yellow watery diarrhœa of *Grat.* is painful, copious, and frequent, preceded by rumblings and cuttings in the abdomen, and nausea. The pain is not relieved by the evacuation, but is by the escape of flatulence. Opposed to this is Hyos., which has similar discharges, *without pain*, often involuntary, and unnoticed in the bed, and is wanting in the extreme offensiveness of those of Ars. and Chin. It is so like one form of diarrhœa frequent in abdominal typhus that the most careless can hardly overlook it as a remedy of prime importance in this most dangerous malady. In such cases the choice will probably be between Ars., China, and Hyos. In Ars. the evacuations are *small*, and perhaps painful, burning, and offensive. In Chin. they are more copious, and in the elements common to the two, less in degree, and Chin. lacks the restlessness already spoken of; while Hyos. is almost the opposite of both in all, except that the three have in common the yellow, watery discharge. In this form of typhus, if the general symptoms, and especially those of the intelligence, delirium, etc., are like those of Hyos., this remedy should certainly be given, and not *soon* changed for *any* other, but for the stronger reasons. To the above may be added Thuja as worthy of attention in these diarrhœas, especially when copious, with gurgling, like that when a full vessel discharges its contents from the bunghole, great prostration, short and difficult breathing, anxiety, intermittent pulse, pressing pain in the back, opposite the epigastrium, and rapid emaciation.

*Gray* or *whitish* watery diarrhœas have Cast., Merc., Phos., and Phos. acid.

Cast. preceded by rumblings, gurglings, croakings, with pinchings and cuttings in the bowels, for the most part in the evening and night; Merc. with cutting and tenesmus; Phos. with great exhaustion and Phos. acid with little or none.

*Purulent* diarrhœas are met by Ars., Bell., Calc., Canth., Chin., Cocc., Kali c., Lach., Lyc., Merc., Puls., Sep., Sil., and Sulph. and some others. The most important of these, in this class, are Ars., Canth., Lach., Lyc., Merc., Puls., Sulph. With Ars. there is a mixture of *blood* and *pus*. Lach. has also mixed pus and blood with gnawing, shooting, cutting pain in a hard swelling in the abdomen. Merc. *chill between*, and flashing heat *during* the stools. Tenesmus characterizes most diarrhœas by this drug, and there is also great uneasiness *before* the stool, and with many cold perspiration *on the face*, anxiety and trembling *before* and heart-burn and bitter eructations *after* the stool. The pains, especially those in the back, and tenesmus are continued *after* the stool. Sulph. has mixed blood, mucus and pus, and the blood is likely to be in streaks. The above brief analysis of the *nature* and *color* of the evacuations in these different examples of diarrhœa is given only as an illustration of the method of proceeding in the first step of an attempt at making a specific prescription.

ODOR.—This may be either simply offensive, or it may be characterized by a specific quality, capable of more specific designation. Of those diarrhœas, which are simply offensive, some are more and others less so. Those in which this characteristic is most intense are met by Arsenicum, Asaf., Carbo v., Graph., Puls., Sec. corn., Sil. and Sulph. Ars. is characterized as like *stinking ulcers* and as putrid. Asaf. as brown and *disgustingly offensive.* Carbo veg. like *putrid flesh.* Graph. is light or brown colored, half digested, thin, and intensely stinking. Sec. corn. has extremely offensive, colliquative diarrhœas. Sil. *small,* liquid, *putrid.* Sulph., on the contrary, is copious and putrid. All the secretions, under the action of Sulphur, are likely to become offensive in the odor. The same is true of the *carbons.* The class of diarrhœas which are less offensive are met by Bry., Calc. c., Cham., Chin., Dulc., Nit. acid., Nux v., Pod., Squill., Staph., Stram. Bry., like spoiled cheese; Calc. carb. and Cham. like *putrid eggs,* that of Cham. being *hot* and *excoriating.* Nit. acid., putrid, with putrid flatulence. Nux v., putrid. Pod., putrid, dark yellow slime. Squill., brown slime expelled in bubbles.

Acid smelling have Calc. c., Cham., Graph., Mag. c., Merc., Rheum, Sep., Sulph. Of these, Calc. and Cham. belong especially to the diarrhœas of children. That of Graph. is accompanied by burning in the rectum. Mag. c., different varieties of diarrhœa of children. Rheum has papescent, acid evacuations, with shuddering, and followed by renewed inclinations and gripings in the bowels. Sep. acid and green, with children.

*Frothy* diarrhœas have for their cure Calc. c., Canth., Coloc., Mag. c., Merc. or Pod., Rhus, Sulph., and Sulph. acid. With Calc. the evacuations are involuntary. Canth., liquid, feculent. Coloc., thin, yellow, and moldy smelling. Mag. c., green and frothy. Merc., dark green. Opium, has fluid, frothy evacuations, with itching burning of the anus and tenesmus. Pod., frothy and slimy. Rhus, thin, yellow, odorless, painless, and involuntary. Sulph., nights, and with tenesmus. Sulph. acid., with burning in the rectum.

*Involuntary* diarrhœas have Arn., Ars., Bell., Bry., Colch., Fer., Hell., Hyos., Lach., Mur. acid., Nat. mur., Nux v., Phos., Phos. acid.,

Rhus, Sec. cor., Staph., Sulph., Verat. Of these the most frequently called for are Ars. With this remedy the evacuation is both involuntary and *unnoticed*. Chin., it is thin, yellowish, and slimy. Phos., Phos. acid., it is pappy, bright yellow, and is passed with a sensation as if wind were about to escape (*Aloes*). Verat has also this last peculiarity of unnoticed evacuation with the escape of wind. Of the other remedies named above, Arn. has involuntary evacuations at *night* in sleep. Bell. and Hyos. both have this variety, as if from paralysis of the sphincter ani. Colch. has watery diarrhœa, the evacuations of which escape without sensation to the patient. Laur. has unnoticed and involuntary evacuations, and in this symptom is very like Bell. and Hyos. It has actual paralysis of the sphincter. Rhus has sudden, thin, yellow, frothy, odorless, and painless, involuntary as from paralysis of the sphincter. Staph. has thin, unnoticed discharges, with sensation as if gas were to escape. Sulph., the stool escapes suddenly and without control; the patient has hardly time to leave the bed.

*Undigested* food, passed with alvine evacuations, is found for the most part in cases which come within our definition of diarrhœa, and which are related to Ars., Bry., Chin., Fer., Merc., Oleand., Phos., Phos. acid., and Pod., and in a less degree to some others. There are cases in which undigested substances are evacuated and which may be subjects for medical interference, which do not come within this scope. With these we are not at present concerned. But in cases which do, how are we to decide which is the right curative? By a reference to the Materia Medica the mention of this symptom is found to be so nearly in the same words, in the record of many of the above medicines, that if this alone be depended on, there can hardly fail to be not a little embarrassment and frequent disappointment. Take three of the principal of them, *i. e.*, those more frequently prescribed and successful than many others, viz.: Ars., Chin., and Ferr., the one word undigested is all, with the first and third, while with Chin. it is added especially at night and immediately after eating. Under Bry., Merc., Phos., Phos. ac., and Pod., the phraseology is the same as with Ars., and there is no additional help from the mention of any circumstance or condition which in any respect characterizes the symptom as manifested by either of these drugs. In the record of Oleander it is said that the food eaten the evening before is passed undigested while it seemed as though wind only was about to escape. If its administration be limited to cases thus characterized, its use can hardly be frequent.

How then are we to proceed? By a careful consideration of the other elements of the case, giving special attention to those which are general or constitutional, *i. e.*, the symptoms outside of the elements of the diarrhœa. For it is never to be forgotten that we are prescribing for the *man*, not merely for that group of phenomena we have, for convenience, consented to call diarrhœa. It is not uncommon that the fact which removes all difficulty and decides beyond doubt the selection of the right remedy is found outside this group. In this statement is an important principle in practical medicine which we hope to elucidate more fully on a future occasion. On this, it will be enough if we can establish the truth in the minds of all that true prescribing can only rest on a thorough analysis of *all* the elements of the case, both general and special, no one excluding others, though, as above stated, one symptom may so far throw light on others as to remove doubt in the choice of a remedy. This is not to say that all symptoms are equally important in their bearing on the selection of a curative, but that none are to be overlooked, for till considered carefully in itself and in its relations, we must be in ignorance of the true value of any, and perhaps of every, symptom. A careful consideration of the constitutional symptoms and general conditions is not limited to cases of undigested evacuations. It is a duty which is integral in every true prescription.

*Acrid* diarrhœas, those in which the evacuations irritate the external parts with which they are brought in contact, are a class too important to be passed without notice. They are related to many drugs in the action of which this quality of the evacuations is evinced in different degrees. The most acrid are from Ars., Chin., Ign., Merc., and Puls. The next in severity are Ant. crud., Cham., Dulc., Fer., Graph., Kali c., Nux vom., Phos., Staph., Sulph., and Verat. And in still less severe are Acon., Alum., Nat. mur., and Sabina. This difference in the intenseness of a symptom is often of great importance and never to be overlooked. With some drugs intenseness seems to characterize most of their actions on the organism, and this goes far at times in individualizing those drugs. Ars. is an eminent instance of this; and no one need fail to distinguish between the painful rawness of the surface around the anus, characteristic of the drug, and the slighter and comparatively insignificant irritation of Acon. Rightly to appreciate this quality of symptoms, and always to give it its just place in a prescription, is an accomplishment of the master, and with him it is an element of great power. It can be cultivated by all, and be carried to a degree the tyro is not likely at first to suspect.

There are, however, other differences in connection with this symptom, expressed in the pathogenesis of some of the above drugs, which are so far our guides, though often we may be left to the significance of general or other special elements of the case. Thus, Ars. has *black*, burning, excoriating evacuations, with restlessness. Merc., *dark green*, with pressure in the abdomen. Puls., soft evacuations in the morning.

The above are the chief elements of analysis of the nature and character of the evacuations in the different forms of diarrhœa. We have next to look at the time, the circumstances, etc., by which attacks are excited or aggravated. And first as to the time: In the morning are Aloes, Ant. crud., Alum., Aur., Amm. carb., Borax, Bov., Bry., Carb. an., Dig., Grat., Iod., Kali carb., Lyc., Mag. c., Mur. acid, Nux v., Phos., Pod., Puls., Sec. cor., Staph., Sulph., Thuja.

With Aloes the evacuations are copious and pappy. Alum., semifluid, preceded by colic. Amm. carb., small, with excoriating and bruised pain in abdomen. Borax, painless, followed by slimy and bloody discharges. Bov., pain in the abdomen like that of ulceration. Carbo an., pinchings in the abdomen, before and after, burning in the anus like fire. Kali c., watery, preceded by colic. Lyc., three to four o'clock, with colic and tenesmus. Mag. c., followed by burning in the anus. Nux v., small, dark, excoriating. Phos., semi-fluid, with rumbling. Puls., soft, excoriating, with smarting. Sec. corn., four o'clock. Staph., after cuttings and nausea. Sulph., at four and six o'clock, and also on rising from bed, the desire is sudden and urgent and admits of no delay. This is characteristic. Thuja, soft.

In the forenoon: Carbo an., Kali c., Kali nit., Mag. c., Mur. acid, Stann., Sulph. Carbo an., soft, green, with colic. Kali c., watery, preceded by rumbling. Mag. c., soft. Stann., soft. Sulph., thin, with pressure in the stomach.

At noon: Alum., Borax, Mag. mur., and Sulph. Alum., semifluid with previous colic. Borax, thin, with rumbling and movements in the abdomen. Mag. mur., severe urgency to stool, which is fluid. Sulph., frothy, feculent, with much flatulence.

In the afternoon: Aloes, Amm. c., Alum., Borax, Carbo an., Dulc., Hell., Kali c., Lyc., Mag. c., Mur. acid, Phos., Stann., Sulph. acid.

Amm. c., first part is hard, the latter soft, with shootings in the anus. Alum., soft and small. Borax, with much flatulence. Carbo an., soft, green, pain in the bowels before the evacuation. Dulc., with flutulence. Kali c., semi-fluid, scanty, with colic, and followed by tenesmus. Phos., semi-fluid, scanty, escaping with force.

Evening: Aloes, Alum, Bov., Carbo an., Dig., Dulc., Indigo, Kali c., Kali nit., Lach., Mang., Merc., Mur. acid, Ol. an., Phell., Stann., and Zinc. Aloes, very thin, deep yellow, with undigested food. Alum., soft, flatulent, with burning in the anus, followed by tenesmus. Dig., with ascarides. Dulc., acid smelling, copious, thin, relieves the pain, while the patient feels weak. Lach., great urgency to stool, with throbbing in the anus after the evacuation. Mang., preceding shootings in the bowels. Mur. acid, severe burning in the anus after the evacuations. Ol. an., soft feces, with cuttings in the bowels before, during, and after the stool, followed by burning in the anus like fire. Stann., with sensation after the evacuation as if there were still more to pass. Zinc, first a little solid, then scanty, soft evacuations.

At night: Arn., Ars., Aur., Bov., Bry., Cast., Caust., Cham., Chel., Chin., Graph., Grat., Kali c., Mag. c., Merc., Nat. carb., Puls., Sil., Sulph., Tabac. Aur., with much burning in the rectum. Bov., with tearing pains in the bowels and tenesmus. Bry., with burning in the anus. Cast., semi-fluid, feces extremely offensive with stinking flatus. Cham., with cuttings in the bowels which double up the patient. Mag. c., before midnight and painless. Puls., unnoticed, watery, in sleep. Sil., painless. Sulph., frequent, fluid, frothy, with tenesmus.

The next element to consider is the exciting cause of the attack. If it be from

Acids.—Ant. crud., thin with pain in the rectum. Ars., Lach., attacks are slight. Phos., acid.

Taking Cold.—Bell., with vomiting. Bry., Caust., Cham., Dulc., watery, at night, with pains in the bowels, in summer, or with prolapsus ani. Nux mos., Nux v., watery. Phos., with cutting and drawing pains in bowels and loins, as far into the thighs. Sulph.

Drinking.—Ars., Caps., of slime. Rhod., painless.

Eating.—Ars., Borax, with rumblings or weakness in the joints and legs, relieved by walking. China, Coloc., with colic after the least nourishment. Fer. mag., Rhod., painless. Verat, after the least ingesta.

FRUIT.—Ars., Chin., Cist., Rhod., with sensation of weakness in the stomach and nausea while walking.

MILK.—Lyc., Nux mos., Sep.

In prescribing for attacks from the above causes, the applicability of those remedies here named, without symptoms, is determined by their general characteristics or by the special analysis and ascertained resemblance of their symptom to those of the individual case. The same principle governs in treating the cases of

INFANTS.—For which Cham., Jalap., Rheum, Senn., and Sulph. acid are more frequently required than other remedies, although it may be remarked of Jalap that its passages are watery, and accompanied with intense cuttings in the bowels; of Rheum, there are mixed feces and slime; of Senn., dark-colored water, with cutting pains also, but less severe than those of Jalap, and more or less flatulent. And also in those of infants while

TEETHING, for which we have Colch., Carbo., Cham., Graph., Merc. sol., Nux mosch., Pod., Sulph. In selecting a remedy from among these, it may help to bear in mind the resemblances and differences of the symptoms of these medicines. Calc. and Graph. are alike in these particulars: both have very offensive discharges, but that of Calc. is yellow, Graph., dark, half digested. Both have acid discharges; that of Graph. is only soft; Calc., thin; Calc. has undigested, hard or thin; Graph., half digested. It is also quite characteristic of Graph. that the discharges are followed by great but *transient* prostration. Calc. and Cham. have much similarity of some symptoms, but the differences of others make the distinction between the two not difficult. Both have the smell of bad eggs, those of Cham. with this property are also excoriating. With Cham. the passages are often green, with Calc. never. The diarrhœa of teething infants, for which Nux mosch. is appropriate, is attended by an indomitable disposition to sleep. The little patient sleeps all the time. The discharges are likely to be very offensive and rather copious. It is a remedy of greater value in teething diarrhœas than is generally supposed. Pod., painful, with grinding of teeth. This, of course, can only occur in cases of the last teeth in the series. Sulph., the discharges are slimy for the most part, brown, green, or white, and often are marked with slight *streaks* of blood.

The above are only a few of the distinguishing symptoms of these drugs, not given as a complete analysis, but only as showing the mode in which distinctions are arrived at in classes of cases where one of a class of similar remedies is to be selected, by which that most unsatisfactory practice of giving one remedy of a class, and, if not successful, another, and so on through the series, may be avoided.

Pregnancy is often attended with obstinate and sometimes fatal diarrhœas. For these cases we may find a remedy in one of the following: Am. carb, Dulc., Hyos., Lyc., Petr., Phos., Sep., Sulph. In these cases, in addition to the careful observation of the elements of the diarrhœa, the constitutional symptoms are to be most rigidly studied, for these, not unfrequently, are decisive of the choice of the remedy. Without a thorough knowledge of these the prescriber must often be quite in the dark as to his curative, and his patient, consequently, in a very unsafe condition. These remarks are equally true of the diarrhœas which arise at

Lying-in.—For these we have Ant. crud., Dul., Hyos., Rheum. There may be cases requiring other drugs, but these can hardly fail of being detected if the analysis and comparison insisted on be faithfully carried out. The above remedies are only named, because so frequently called for, that they may claim our first attention in cases where the characteristics of other drugs are not prominent. They are never to be given merely because named here, or elsewhere, as possibly appropriate in this class of cases.

It may facilitate the treatment of diarrhœas to study them in *groups*. Thus Ars., Chin. and Fer. have the closest relationship. In these Ars. occupies one extreme and Chin. the other, Fer. falling between. In the element of *pain*, Ars. has extreme severity, Fer. less, and Chin. less still. In that of copiousness, with the exception of yellow watery, in which Ars. represents the scanty, the same relation obtains. It may be borne in mind that cases of an obstinate character sometimes occur where those remedies act beneficially in succession. Thus, in cases in which Fer. has followed Chin. with benefit, but has not proved sufficient for a complete cure, Ars., if at all appropriate, seldom fails to effect that result. Verat. may be added to this group in the study of watery diarrhœas, and in the elements of copiousness and pain takes place next to Ars.

Another most important group is represented by Ars., Squill., Graph., and Nux vom., viz.: the dark, fluid, offensive, and painful. In these elements the four remedies agree. They differ, however, in so many of their symptoms that there can be no serious difficulty in selecting the right for a given one, if it be borne in mind that Ars. among these has the most copious evacuations; Nux v. the least, and always *small.* The pain of Ars. and Squill. is in the bowels, Ars. the most severe; those of Nux v. and Graph. in both the bowels and back, Nux the most severe, with this further difference, that the pain of Nux is higher in the loins, Graph. in the sacral region. With Nux the pain is drawing and is relieved by the evacuation; Graph., pressing and continued after. It may not be amiss before leaving this group to say that Nux vom. has been too much neglected in the treatment of diarrhœas. The frequent successful use of the drug in constipation may have so occupied the minds of prescribers as to limit, in their apprehension, its usefulness to cases of this sort. This is a great mistake or misfortune. It is scarcely less important as a remedy for diarrhœa. It has been the specific in many epidemics, and at other times, through whole seasons, it has been oftener called for and successful than any other drug.

Ars., Gamboge, Jalap., and Senna in extremely painful diarrhœas. The characteristics of these remedies and their distinctions, except Gamb., have already been noticed, and it may be sufficient for this to say that it resembles Arsenic more than either of the others, but with the evacuations of Gamb. there is much disposition to *tenesmus*, while with Ars. there is less.

Arn., Lach., Merc., and Sulph. in purulent diarrhœas. Arn. has bloody and purulent discharges. Lach. has similar evacuations, with gnawing, shooting, cutting pains, with hard swelling of the abdomen, or with discharge of mucus and scanty menses. Sulph., a mixture of blood, mucus, and pus.

Nux mos., Sec. corn., Verat, in cases, with comatose *sleep.* These three remedies are each characterized by profound and constant sleep. The kind of *sleep* is very similar in the three, very quiet and undisturbed, but the conditions out of which it grows are very different and not difficult of distinction. With Nux mos., the symptom arises from *exhaustion of the brain power* especially. There is still sufficient to admit of the patient being aroused without great difficulty, but not to sustain a continued attention to external objects. The patient falls asleep again immediately and continues to sleep till roused by the attendants. The affection is less profound than that of the other two remedies, and generally less dangerous. Sec. corn. is opposed to Nux mos. in this, that its coma seems to rest on a *general* exhausted vital force, in which that of the brain participates, or of which its exhaustion is a part, all the organs being similarly affected, the tendency being to a rapid extinction of life unless the downward progress be speedily arrested. The patient is roused with difficulty and then immediately falls off again, being wholly unable to give attention to external objects for the shortest time. Verat. is related to a condition quite different from both, viz.: that which just precedes the effusion of serum into the cavities of the brain or the early stage of effusion. In such cases Verat. is often very efficacious. If the patient be aroused he shows that he is disturbed and complains. Any interference is painful to him, till he passes the point in insensibility at which he ceases to regard the presence or acts of his attendants, and beyond which all remedies are too likely to fail to relieve.

Aloes, Pod., and Rheum, as related to the class of feculent diarrhœas, have been already sufficiently treated of, though it may be said of Aloes, in addition, that its evacuations are often preceded by much rumbling and movement of flatus in the bowels, flatulent distention, and colic. Not unfrequently these rumblings and movements are, after a night's sleep, first manifested on the patient's first stepping out of bed in the morning. Or they are especially, at evening, and if the flatus escapes it is of the most offensive odor.

It does not come within the plan of the present paper to consider the diarrhœas which are at times concomitants of other more important affections, as of typhus fever, phthsis, etc., further than to remark that an intelligent treatment of them involves, in addition to the analysis of their elements here inculcated, a careful study of the characteristics of the malady, in the elements of which, alone, it often happens the clew can be found to the remedy for this as well as other symptoms of the case. This should never be forgotten. It is a necessity in every case, perfectly apparent in the principle stated, that we treat patients and not diseases. Least of all can we isolate an element of a case and treat that, and claim in such practice the consideration of scientific and able physicians.

